# Therapeutic Effect of Rapamycin on Aortic Dissection in Mice

**DOI:** 10.3390/ijms21093341

**Published:** 2020-05-08

**Authors:** Makiko Hayashi-Hori, Hiroki Aoki, Miho Matsukuma, Ryohei Majima, Yohei Hashimoto, Sohei Ito, Saki Hirakata, Norifumi Nishida, Aya Furusho, Satoko Ohno-Urabe, Yoshihiro Fukumoto

**Affiliations:** 1 Division of Cardiovascular Medicine, Department of Medicine, Kurume University School of Medicine, Kurume, Fukuoka 830-0011, Japan; hayashi_makiko@med.kurume-u.ac.jp (M.H.-H.); majima_ryouhei@med.kurume-u.ac.jp (R.M.); hashimoto_youhei@med.kurume-u.ac.jp (Y.H.); itou_souhei@med.kurume-u.ac.jp (S.I.); hirakata_saki@med.kurume-u.ac.jp (S.H.); nishida_norifumi@med.kurume-u.ac.jp (N.N.); furushyou_aya@med.kurume-u.ac.jp (A.F.); urabe_satoko@kurume-u.ac.jp (S.O.-U.); fukumoto_yoshihiro@med.kurume-u.ac.jp (Y.F.); 2 Cardiovascular Research Institute, Kurume University, Kurume, Fukuoka 830-0011, Japan; 3 Kurume University School of Medicine, Kurume, Fukuoka 830-0011, Japan; miho.apple.0112@icloud.com

**Keywords:** aortic dissection, mTOR, rapamycin, smooth muscle cells, inflammation

## Abstract

Aortic dissection (AD) is a serious clinical condition that is unpredictable and frequently results in fatal outcome. Although rapamycin, an inhibitor of mechanistic target of rapamycin (mTOR), has been reported to be effective in preventing aortopathies in mouse models, its mode of action has yet to be clarified. A mouse AD model that was created by the simultaneous administration of β-aminopropionitrile (BAPN) and angiotensin II (AngII) for 14 days. Rapamycin treatment was started either at day 1 or at day 7 of BAPN+AngII challenge, and continued throughout the observational period. Rapamycin was effective both in preventing AD development and in suppressing AD progression. On the other hand, gefitinib, an inhibitor of growth factor signaling, did not show such a beneficial effect, even though both rapamycin and gefitinib suppressed cell cycle activation in AD. Rapamycin suppressed cell cycle-related genes and induced muscle development-related genes in an AD-related gene expression network without a major impact on inflammation-related genes. Rapamycin augmented the activation of Akt1, Akt2, and Stat3, and maintained the contractile phenotype of aortic smooth muscle cells. These findings indicate that rapamycin was effective both in preventing the development and in suppressing the progression of AD, indicating the importance of the mTOR pathway in AD pathogenesis.

## 1. Introduction

Aortic dissection (AD) is one of the most serious forms of cardiovascular disease. The abrupt disruption of the intima and media occurs with severe chest and back pain that is preceded by few, if any, symptoms [1]. After onset, AD causes further destruction of the aortic wall by tearing the medial layer apart, resulting in deterioration of the heart and distal organs. Untreated AD involving the ascending aorta (a Stanford Type A dissection) results in 75% mortality within 2 weeks [2]. When AD does not involve the ascending aorta (a Stanford Type B dissection), the acute mortality is approximately 10% [3]. According to the difference in the mortalities, current recommendations for therapeutic options are emergency surgery for Type A and medical therapy for Type B dissection [4]. In addition, recent advances in new devices and techniques for stent-grafting are making thoracic endovascular aortic repair a new therapeutic option. Even with the advancement in therapeutic options, patients suffer high mortality in the acute phase and long-term complications due to the progressive destruction of aortic wall in the chronic phase of AD [5]. Although new therapeutic options are much awaited, effective medical therapy is not available due to the lack of knowledge of AD molecular pathogenesis.

Several gene mutations are known to predispose individuals to AD [6]. The culprit genes for the syndromic aortic diseases include *FBN1* for Marfan syndrome, transforming growth factor β (TGFβ)-related genes for Loeys-Dietz syndrome, and *COL3A1* for type IV Ehlers-Danlos syndrome. The genes for the non-syndromic forms include those for contractile proteins of smooth muscle cells (SMCs), metabolism of SMCs, and extracellular matrix (ECM) metabolism. These hereditary forms of AD predisposition underscore the importance of SMCs and ECM metabolism in AD pathogenesis. The importance of SMCs in AD pathogenesis has also been demonstrated in animal models of AD, as we and others have identified molecules in SMCs that are protective against AD, including tenascin C [7], Stat3 [8], Akt2 [9], and Sirt1 [10]. In addition, inflammatory response is proposed to be important for AD [11,12,13,14]. The link between the inflammatory response and the altered function of SMCs in AD may be explained by the plasticity of the SMC phenotype in pathologic conditions [15]. In response to changes in their environment, SMCs may lose the contractile phenotype and acquire the synthetic phenotype that is characterized by the expression of secretory molecules, including cytokines and ECM molecules, as well as proliferative capacity. Indeed, acquisition of the SMC synthetic phenotype has been demonstrated both in human AD [16] and in animal models of AD [11,15,17,18]. Consistently, cell cycle activation was also reported in human AD [16,19], and we have demonstrated that the cell cycle activation precedes the proinflammatory response during the development of AD in mice [11].

Regarding the therapeutic target for AD, treatment of mice with rapamycin, an inhibitor of mechanistic target of rapamycin (mTOR), was reported to prevent AD in mice with postnatal disruption of *Tgfbr2* in SMCs, a model of Loeys-Dietz syndrome [17,20], and in another AD model that was induced by administration of β-aminopropionitrile (BAPN). The beneficial effect of rapamycin was associated with suppression of the proliferative response and recovery of aortic contractility in the Loeys-Dietz syndrome model. However, it is unclear whether these findings are specific to the aortopathy due to the specific disruption of *Tgfbr2* in SMCs. Furthermore, it is unknown whether rapamycin is effective in treating AD after it develops. In this study, we examined the effects of rapamycin and gefitinib, an inhibitor of growth factor signaling, in a mouse AD model created by the administration of BAPN and angiotensin II (AngII) [21]. To better understand the molecular pathogenesis of AD and the effect of rapamycin, we analyzed gene expression networks and intracellular signaling in the AD model and in cultured SMCs.

## 2. Results

### 2.1. Effect of Cell Cycle Inhibitors on AD

**Figure 1:**
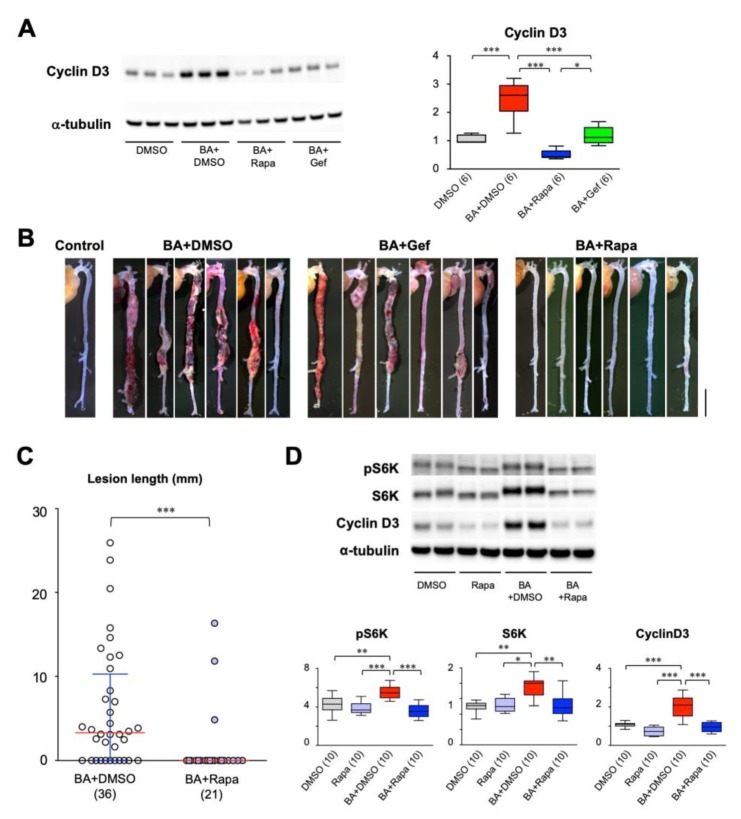
Effect of cell cycle inhibitors on aortic dissection (AD). (**A**,**B**) Effects of rapamycin and gefitinib on the expression of cyclin D3 (**A**) and macroscopic findings (**B**) in a murine model of AD. Scale bar: 5 mm. (**C**,**D**) Quantitative analysis of the effect of rapamycin on AD lesion length (**C**), p70 S6 kinase (S6K), activated (phosphorylated) S6K (pS6K), and cyclin D3 (**D**). The figures in parentheses indicate the numbers of independent observations. * *p* < 0.05, ** *p* < 0.01, *** *p* < 0.001. Among the multiple experimental groups, all comparisons between two groups were tested and for simplicity only those with statistically significant differences are indicated.

We previously reported that cell cycle activation precedes AD development. This finding prompted us to test the effect of cell cycle inhibitors, namely gefitinib, an inhibitor of epidermal growth factor receptor (EGFR), and rapamycin, an inhibitor of mTOR, on AD. We created a mouse model of AD by continuous infusion of angiotensin II and β-aminopropionitrile (AngII + BAPN) [21]. In this model, AD started to develop around day 7 of AngII + BAPN infusion and further progressed during the observational period of 14 days of AngII + BAPN infusion. The time course of this AD model allowed us to examine the effect of cell cycle inhibitors on both the development and progression of AD. We examined the expression of cyclin D3 as an indicator of cell cycle activation (Figure 1A). Both gefitinib and rapamycin suppressed cyclin D3 expression after 3-day infusion of BAPN + AngII before AD development, suggesting that the EGFR and mTOR pathways are involved in cell cycle activation during AD development. Rapamycin showed more effective suppression of cyclin D3 than did gefitinib, albeit both suppressed cyclin D3 to levels comparable to that of the vehicle-treated group. Macroscopic examination revealed that gefitinib had no obvious effect on AD. On the other hand, rapamycin showed a striking suppression of AD development after 14-day BAPN + AngII challenge (Figure 1B, Table 1, *p* < 0.01 by Fisher’s exact test) without significant changes in systolic blood pressure, pulse rate or body weight (Figure S1). Therefore, we decided to focus on the effect of rapamycin on AD either in the preventive protocol or in the therapeutic protocol (Figure S2). In an experiment with an increased number of mice with the preventive protocol, the incidence of AD was 69.4% in the vehicle (dimethylsulfoxide; DMSO) group (BA+DMSO) and 20.0% in the rapamycin group, demonstrating significant suppression of AD by rapamycin (Table S1, *p* < 0.01 by Fisher’s exact test). Rapamycin treatment significantly suppressed AD severity as determined by the lesion length [8,21] (Figure 1C). Western blot analysis showed that rapamycin reduced cyclin D3 expression, as well as the levels of total and phosphorylated p70 S6 kinase without changes in the ratio of the phosphorylated form to the total protein (Figure 1D).

### 2.2. Rapamycin-Sensitive Cell Types in AD

**Figure 2:**
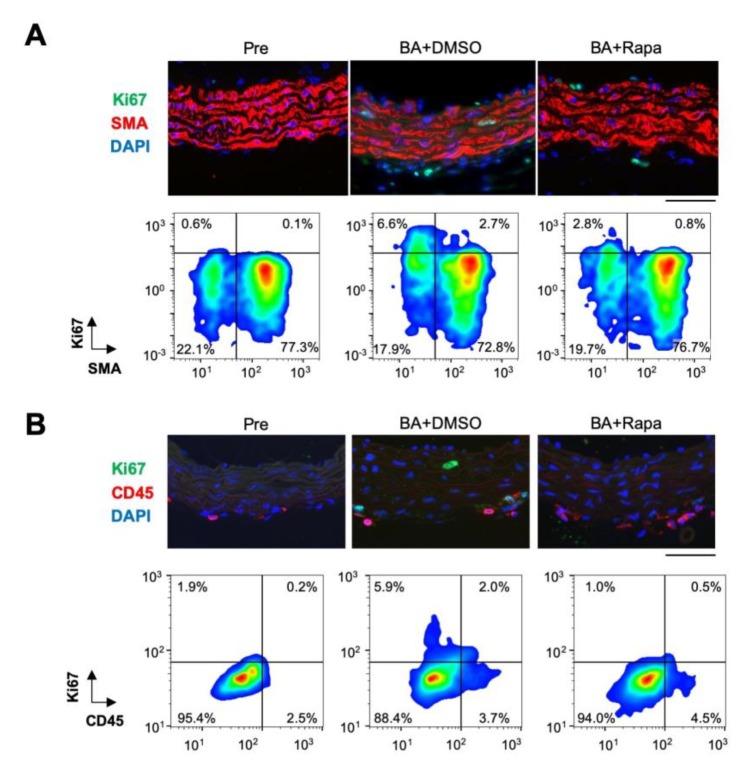
Effect of rapamycin in aortic tissue. Immunofluorescence staining of the aortic tissue for Ki67 and SMA (**A**) or CD45 (**B**) with nuclear 4’,6-diamidino-2-phenylindole (DAPI) staining, before (Pre) or after 3 days of BAPN + AngII challenge with vehicle (BA+DMSO) or rapamycin (BA+Rapa) treatment. Representative immunofluorescence images (upper panels) and scattergrams of imaging cytometry (bottom panels) are shown. The percentages of cell populations are indicated in the scattergrams of the quadrants as arbitrarily determined by the thresholds for the signal intensities of Ki67 (**A**,**B**), SMA (**A**) and CD45 (**B**). The thresholds are set constant for Pre, BA+DMSO, and BA+Rapa in a given double staining. Scale bars: 50 µm.

To assess the effect of rapamycin in a cell type-specific manner, we performed immunostaining for Ki67, a marker of cell proliferation, and smooth muscle α-actin (SMA), a marker SMCs (Figure 2, Figure S3). BAPN + AngII challenge induced Ki67 expression in both SMA-positive and -negative subpopulations, and this expression was suppressed by rapamycin in both. We also examined the expression of Ki67 with CD45, a marker of inflammatory cells (Figure S3). BAPN + AngII challenge induced Ki67 expression in both CD45-positive and -negative subpopulations, and this expression was suppressed by rapamycin in both. We also examined the expression of Ki67 with specific inflammatory cell markers, namely B220 for B cells, CD3 for T cells, Iba-1 for macrophages, and Ly-6G for neutrophils (Figure S4). Among these inflammatory cells, Ki67 expression overlapped with some Iba-1-positive cells in aortic tissue with BAPN + AngII challenge. Since the numbers of inflammatory cells were small at day 3 of BAPN + AngII, we could not quantitatively assess the Ki67-positive cells or the effect of rapamycin on other cell types. These results suggested that BAPN + AngII activated the cell cycle in a rapamycin-sensitive manner in various cell types: SMA-positive SMCs, CD45-positive inflammatory cells, and the double-negative cell population.

### 2.3. AD-Related Gene Expression Network

**Figure 3:**
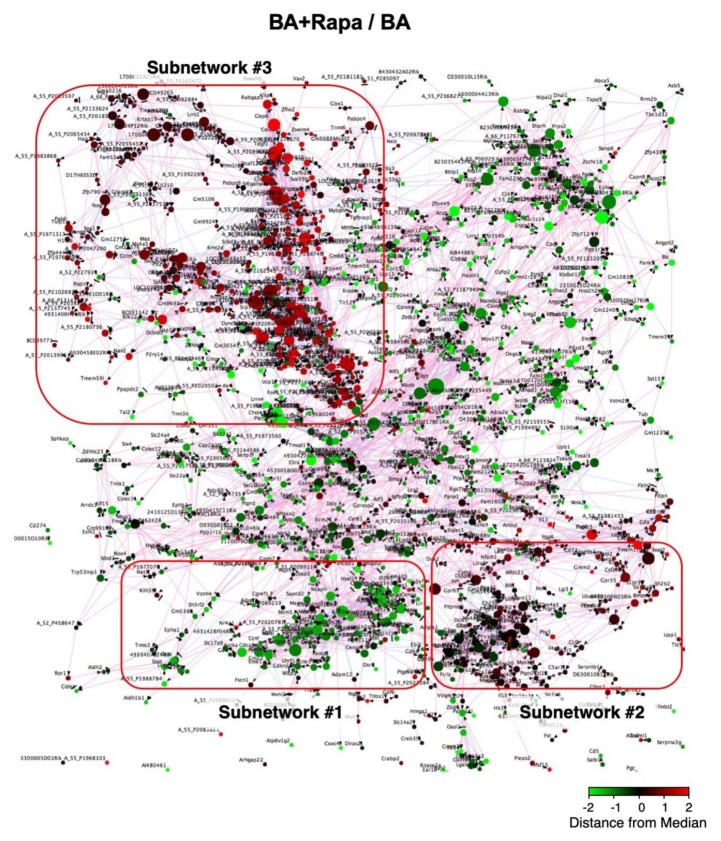
Gene expression network in AD. Organic plot of the Bayesian network analysis for gene expression. Each node represents a single gene that is color coded for induction (red) or suppression (green) by rapamycin in the presence of BAPN + AngII challenge. The genes are connected by red or blue edges that represent positive or negative correlations, respectively, between a pair of genes on both sides of the edge. The higher the correlations are, the closer the genes are plotted. Three clusters of the nodes represent the groups of genes making tightly coupled subnetworks (#1, #2, and #3) for their expressions.

To better understand the effect of rapamycin on AD, we performed transcriptome and gene expression network analyses (Figure 3, Figures S5–S8). The Bayesian network analysis of the AD-related genes revealed several subnetworks of genes that were co-regulated in response to BAPN + AngII challenge and rapamycin treatment (Subnetworks #1, #2, and #3 in Figure 3). Gene annotation enrichment analysis revealed that Subnetwork #1 was enriched for cell cycle-related genes, Subnetwork #2 was enriched for inflammation-related genes, and Subnetwork #3 was enriched for genes related to cell size regulation and muscle development (Table 2, Table S2). BAPN + AngII challenge caused activation of Subnetworks #1, #2, and #3 (Figure S8). When the effect of rapamycin was examined, BAPN + AngII-mediated activation of Subnetwork #1 was reversed, Subnetwork #2 was unchanged, and Subnetwork #3 was further activated (Figure 3). These results suggested that rapamycin reversed cell activation by BAPN + AngII without having a major impact on the inflammatory response. In addition, rapamycin enhanced the activation of genes that are related to cell size regulation and muscle development.

### 2.4. Effect of Rapamycin on Protein Expression in the Aorta

**Figure 4:**
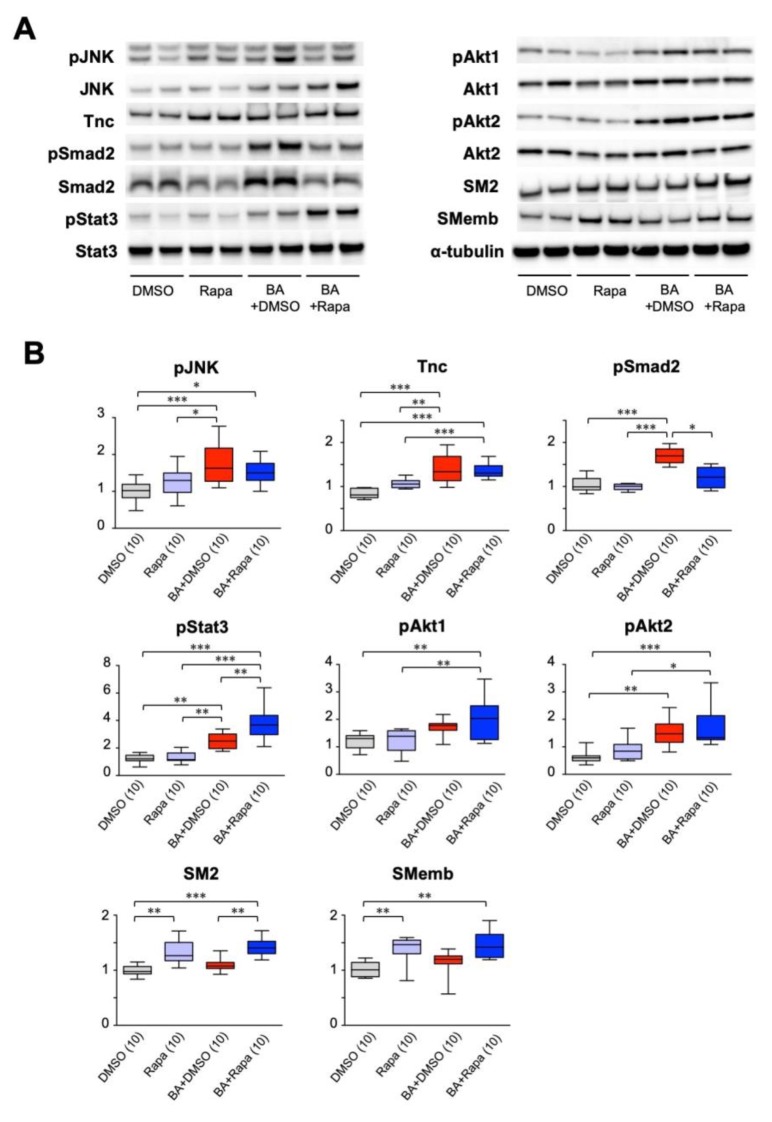
Effect of rapamycin on cellular signaling in AD. Effect of rapamycin on protein expression in the AD model with or without 3 days of BAPN + AngII challenge. Mice were treated with vehicle (DMSO) or rapamycin without or during the BAPN + AngII challenge (BA). Representative images for Western blotting (**A**) and quantitative analysis of the selected proteins (**B**) are shown. The figures in parentheses indicate the numbers of independent observations. * *p* < 0.05, ** *p* < 0.01, *** *p* < 0.001. Among the multiple experimental groups, all comparisons between two groups were tested and for simplicity only those with statistically significant differences are indicated.

AD pathogenesis involves an inflammatory response, and the gene-expression network analysis above suggested the importance of the growth response for the protective effect of rapamycin. We therefore examined the expression and activation of molecules in inflammatory and growth responses that are reported to participate in the mechanism of AD (Figure 4). Inflammatory and tissue repair signal mediators, namely activation (phosphorylation) of Jnk [22], and the expression of tenascin C [7] was induced by BAPN + AngII challenge. The cellular contents of the phosphorylated and total Smad2, which is involved in tissue repair response [6,21,23], were upregulated by BAPN + AngII and suppressed rapamycin, albeit the ratio of the phosphorylated form to the total proteins did not change. BAPN + AngII challenge activated signal mediators for cell survival and growth, namely Stat3 [8,11] and Akt1 and Akt2 [9,24], and this activation increased in the presence of rapamycin. We also examined the expression of myosin heavy-chain isoforms to assess the phenotypic modulation of SMCs; namely SM2, a marker of the contractile phenotype, and SMemb, a marker of the synthetic phenotype. Rapamycin treatment augmented the expression of SM2, and paradoxically also augmented the expression of SMemb.

### 2.5. Effect of Rapamycin on Stat3, Akt1, and Akt2 in SMCs

**Figure 5:**
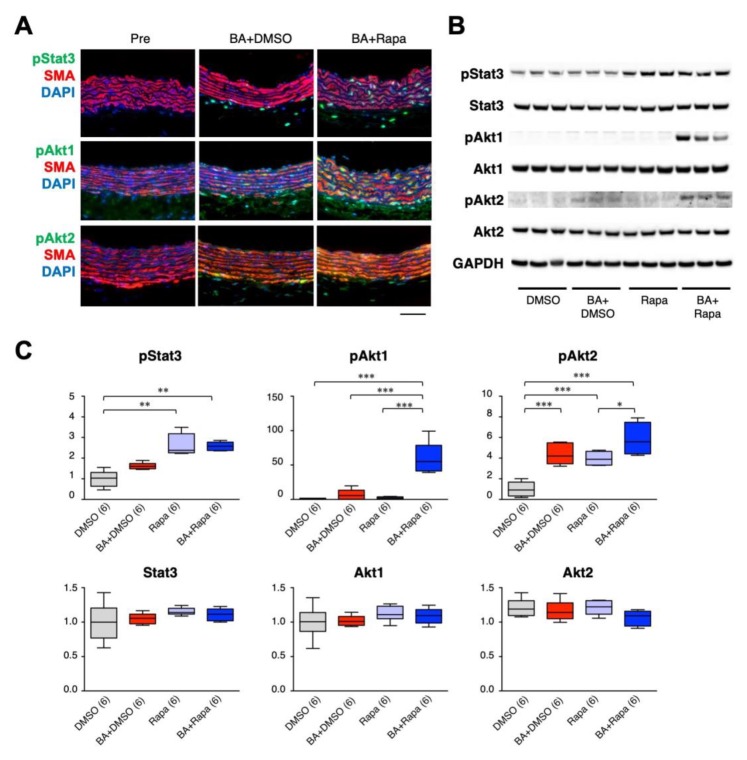
Effect of rapamycin on cellular signaling in SMCs. Effect of 3 days of BAPN + AngII (BA) and rapamycin on pStat3, pAkt1, and pAkt2 in aortic tissue (**A**) or in SMCs in culture (B, C). (A) Representative immunofluorescence images of triple staining for SMA (red), nuclear DAPI (blue) staining, and pStat3, pAkt1, or pAkt2 (green). Scale bar: 50 µm. (**B**) Representative images of Western blotting for the indicated proteins. Glyceraldehyde-3-phosphate dehydrogenase (GAPDH) served as an internal loading control. (**C**) Quantitative analysis of the indicated proteins. The figures in parentheses indicate the numbers of independent observations. * *p* < 0.05, ** *p* < 0.01, *** *p* < 0.001. Among the multiple experimental groups, all comparisons between two groups were tested and for simplicity only those with statistically significant differences are indicated.

To further characterize the activation status of Stat3, Akt1, and Akt2 in aortic tissue, we performed double immunofluorescence staining for pStat3, pAkt1, and pAkt2 along with SMA, a marker of SMCs (Figure 5A, Figure S9 and S10). BAPN + AngII challenge caused pStat3 activation predominantly in adventitial non-SMCs, and additional rapamycin treatment augmented pStat3 in medial SMCs. BAPN + AngII caused activation of Akt1 and Akt2 mainly in medial SMCs, which were further augmented by the rapamycin treatment. We next examined if the rapamycin-mediated signal augmentations were intrinsic to SMCs, using aortic SMCs in culture (Figure 5B,C). Stat3 was activated by rapamycin alone but not by BAPN + AngII. Akt1 was activated only by the combination of BAPN + AngII and rapamycin, whereas Akt2 was activated by either BAPN + AngII or rapamycin, and its activation was further augmented by the combination of BAPN + AngII and rapamycin. These findings partly recapitulated the in vivo responses of Stat3, Akt1, and Akt2 in AD, and indicated the presence of SMC-intrinsic regulatory mechanisms for Akt1 and Akt2.

### 2.6. Effect of Rapamycin on AD Progression

**Figure 6:**
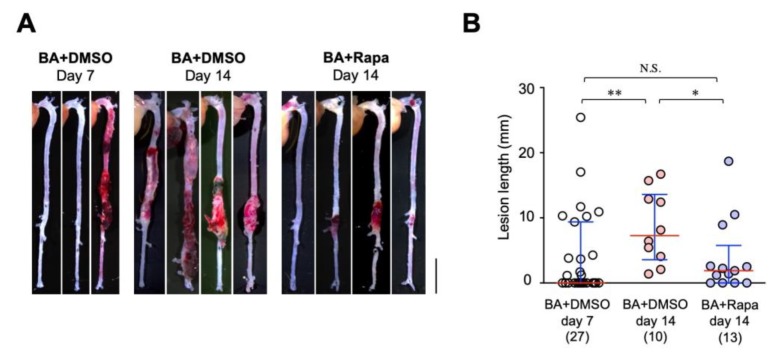
Therapeutic effect of rapamycin on AD. (**A**) Representative macroscopic findings at the indicated periods of BAPN + AngII infusion (BA) with vehicle (DMSO) or rapamycin (Rapa). Scale bar: 5 mm. (**B**) Quantitative analysis of the AD lesion length in the indicated experimental groups. The figures in parentheses indicate the numbers of independent observations. * *p* < 0.05, ** *p* < 0.01, *** *p* < 0.001.

The onset of AD is unpredictable and prevention of AD is very difficult, if not impossible. Therefore, therapeutic approaches can be implemented only in those with AD or at very high risk for AD. Based on this clinical situation, we tested if rapamycin treatment was effective after starting BAPN + AngII challenge. We first evaluated the time course of the BAPN + AngII model. At 7 days of BAPN + AngII challenge, the incidence of AD was 48.1% and the mean lesion length was 4.11 ± 1.25 mm (mean ± standard errors, *n* = 27, Table 3, Figure 6). We made a comparison between vehicle treatment and rapamycin treatment from day 7 to day 14 in the BAPN + AngII model (Figure S1, Table 3, Figure 6). At day 14 of BAPN + AngII challenge, the AD incidence was 100% for the vehicle group and 69.2% for the rapamycin group (*p* = 0.32 by Fisher’s exact test). The severity of AD as assessed by the lesion length was 8.53 ± 1.76 mm (mean ± standard errors, *n* = 10) for the vehicle group and 3.84 ± 1.55 mm (*n* = 13) for the rapamycin group (*p* < 0.05, Table 3, Figure 6), indicating that rapamycin can ameliorate the progression of AD after the start of the BAPN + AngII challenge.

## 3. Discussion

In this study, we demonstrated that rapamycin treatment was effective not only in preventing AD development but also in suppressing the worsening of AD once it began. The transcriptome analysis indicated that rapamycin selectively suppressed the cell cycle-related gene subnetwork without having a major impact on the expression of the inflammation-related subnetwork. Cell cycle activation in AD was confirmed at the protein and tissue levels, and was suppressed by rapamycin, indicating the importance of the rapamycin-sensitive mTOR pathway in cell cycle activation in AD. Rapamycin also activated the subnetwork of genes involved in muscle development. Consistently, expression of SM2, a marker of the contractile phenotype of SMCs, was preserved in the presence of rapamycin. In addition, the AD-suppressive effect of rapamycin was associated with the activation of molecules that are proposed to protect aortic tissue from AD, including Akt2 [9] and Stat3 [8] in SMCs.

A therapeutic effect of rapamycin on aortopathies has been reported in mice with *Tgfbr2* disruption in postnatal SMCs, a model of Loeys-Dietz syndrome [23]. The AD phenotype in Loeys-Dietz syndrome was recapitulated in the mouse model, concomitant with aberrant activation of the mTOR pathway, dysregulated cell proliferation, and loss of the SMC contractile phenotype, all of which were restored by rapamycin. Rapamycin also displayed a beneficial effect in a BAPN-induced AD model [25], although the detailed mechanism was not shown. In addition, rapamycin ameliorates abdominal aortic aneurysm in animal models [26,27,28,29], where its effect has been attributed to the suppression of inflammatory responses. On the other hand, activation of the mTOR pathway was observed in human aortic tissue in the presence of a bicuspid aortic valve, a clinical situation prone to aortic dissection, even though the tissue did not show obvious inflammation [30,31]. In our study, rapamycin showed little effect on the subnetwork of inflammation-related genes, and had no effect on the activation of Jnk, an inflammatory signaling molecule, in the AD model at day 3 of BAPN + AngII challenge. The difference between the previous reports on AAA and the current finding on AD may be due to that experimental AAA was created by inducing inflammation at the beginning, while the cell cycle activation preceded the proinflammatory response in the early stage of AD [11]. The effect of rapamycin was observed predominantly as a suppression of cell cycle in this study, perhaps because we observed the molecular phenotypes at the early time point of day 3 after the BAPN + AngII challenge. It is possible that if the molecular changes were observed at the later stage of AD, rapamycin may show the suppression of inflammatory response. Indeed, a previous study showed that rapamycin suppressed the inflammatory infiltrates after the development of AD [25]. These findings suggest that rapamycin may not ameliorate AD by suppressing inflammation, at least in the current experimental condition.

Rapamycin showed reciprocal suppression of genes related to the cell cycle and the activation of muscle development in aorta. The activation of cell cycle-related genes is consistent with previous reports in human AD samples [19] and in animal models [11,23], suggesting the importance of cell proliferation in AD pathogenesis. However, we found that gefitinib did not show a therapeutic effect on AD, even though it suppressed cyclin D3 expression to a level comparable to that of normal aorta. This finding suggests that a cell proliferative response per se may not be directly involved in AD pathogenesis. Alternatively, a gefitinib-sensitive mechanism may contribute to the proliferative response only partially, as suggested by the more prominent suppression of cyclin D3 by rapamycin than by gefitinib in the presence of BAPN + AngII challenge. If this is the case, incomplete suppression of the proliferative response may explain the absence of a therapeutic effect of gefitinib on AD.

Rapamycin-induced activation of muscle development-related genes and maintenance of the high expression of SM2, a marker of the contractile phenotype of SMCs, suggested that rapamycin preserved SMC contractility. Maintenance of the SMC contractile phenotype is a proposed mechanism for the rapamycin-mediated suppression of aortopathy in mice with SMC-specific postnatal disruption of *Tgfbr2* [23]. As for the regulation of SMC phenotype, the phosphatidylinositol 3-kinase (PI3-K)/Akt pathway is reported to mediate SMC differentiation by insulin-like growth factor-1 (IGF-1) [32]. In addition, the effect of rapamycin in maintaining the contractile phenotype by feedback activation of the PI3-K/Akt2 pathway has been demonstrated in cultured SMCs [33]. Consistently, we found that BAPN + AngII and rapamycin showed synergistic activation of Akt1 and Akt2 in cultured SMCs and in the mouse AD model. It has been reported that Akt2 confers protection against AD [9], and thus enhanced activation of Akt2 by rapamycin may contribute to the beneficial effect of rapamycin on AD. 

Another potential mechanism by which rapamycin protects the aorta is the activation of Stat3 in SMCs. Stat3 activation is reported to promote SMC survival and growth [34,35] and to drive SMCs to the synthetic phenotype [36]. In the context of AD, we previously demonstrated that SMC-specific activation of Stat3 reinforced aortic walls and protects the aorta by promoting the secretion of soluble factors that activate adventitial fibroblasts and collagen deposition [8], consistent with the notion that Stat3 promotes the synthetic SMC phenotype [36]. However, SMC-specific Stat3 activation resulted in higher expression of SM2 [8], suggesting that it maintains the SMC contractile phenotype in the aorta in vivo. Furthermore, the synergistic activation of Stat3 by BAPN + AngII and rapamycin was observed in the aorta, but not in cultured SMCs. These findings demonstrate the complex interplay among AD-inducing external stimuli, the activation of intracellular signaling, and SMC phenotypic regulation, which in concert determine the phenotype of AD. 

Our findings indicate that rapamycin is effective in preventing the development and worsening of AD. Considering the unpredictable nature of AD in people without known high-risk backgrounds, a therapeutic effect after AD development is important. On the other hand, for those with high-risk factors for AD, such as bicuspid aortic valve or familial aortopathies including Marfan syndrome and Loeys-Dietz syndrome, preventive intervention is desirable. In this regard, rapamycin may be a promising therapeutic agent before and after AD development. Since the rapamycin-sensitive functions of mTOR are quite diverse [37,38,39], including, but not limited to, cell proliferation, protein synthesis, energy metabolism, autophagy, and cell death, much more study is required to fully characterize the molecular pathway of aortic wall protection against AD. 

## 4. Materials and Methods 

### 4.1. Animal Experiments

All animal protocols were approved by the Animal Experiments Review Boards of Kurume University. All mice were fed normal chow and allowed access to freely available drinking water unless otherwise stated. All of the animal experiments were carried out using male mice aged 11–14 weeks because AD predominantly affects men [40]. AD was induced by simultaneous administration of BAPN (150 mg/kg/day, Sigma-Aldrich, St. Louis, MO, USA) and AngII (1000 ng/kg/min, Peptide Institute, Osaka, Japan) using osmotic minipumps (Alzet model 1002, DURECT, Cupertino, CA, USA) [21]. Two pumps, one for BAPN and another for AngII, were implanted while the mice were under anesthesia with 2% isoflurane. Gefitinib (100 mg/kg/day in 100 μL DMSO, Cayman Chemical, Ann Arbor, MI, USA), rapamycin (2 mg/kg/day in 100 μL DMSO, LC Laboratories, Woburn, MA, USA), or vehicle (100 μL DMSO) was intraperitoneally injected once a day. The dose of gefitinib was determined according to the previous study [41]. The dose of rapamycin was empirically determined in a preliminary study in which 1, 2, 4, or 8 mg/kg/day of rapamycin was tested in the mouse AD model. The dose of 2 mg/kg/day was chosen, as it suppressed cyclin D3 expression and the AD phenotype without affecting the white blood cell count in peripheral blood. For the preventive intervention, administration of rapamycin, gefitinib, or DMSO was started on the day of the implantation of BAPN and AngII pumps (Figure S1). For the therapeutic intervention with rapamycin, intraperitoneal injection of rapamycin (2 mg/kg/day in 100 μL DMSO) or vehicle (100 μL DMSO) was started on day 7 and continued to day 14 of BAPN + AngII challenge. The mice were killed by pentobarbital overdose at the indicated time points to collect tissue samples (Figure S1). BAPN + AngII challenge or rapamycin treatment did not affect systolic blood pressure, pulse rate, or body weight (Figure S2) The aortic tissue was excised either immediately for protein and mRNA expression analysis, or after perfusion and fixation with 4% paraformaldehyde in phosphate-buffered saline (PBS) at physiological pressure, for morphological and histological analyses. To analyze protein and mRNA expression, a 10 mm length of aorta was excised above the branching point of the right renal artery, quickly frozen in liquid nitrogen, and stored at −80 °C until sample extraction. The severity of AD was assessed by the extent and location of aortic wall destruction as previously described [21], because the extent of aortic destruction is a major determinant of the clinical course [1]. Briefly, the lesions with aortic wall destruction due to AD were defined by a diameter at least 1.5-fold greater than the reference diameter (Figure S2). Due to the differences in the reference diameters, the AD lesion lengths were determined in separate segments of aorta: arch, thoracic descending, suprarenal, and infrarenal.

### 4.2. Histological Analysis

We performed immunofluorescence staining on 5-μm sections, using antibodies to Ki67 (Abcam #ab16667), smooth muscle α-actin (SMA; Sigma-Aldrich, #A5228, St. Louis, MO, USA), CD45 (BioLegend #103101, San Diego, CA, USA), phospho-Stat3 (Cell Signaling Technology #9145, Danvers, MA, USA), phospho-Akt1 (Cell Signaling Technology #4060), phospho-Akt2 (Abcam #ab38513, Cambridge, UK), B220 (BD #553086, Becton Dickinson, Franklin Lakes, NJ, USA), CD3 (Abcam #ab11089), Iba-1 (Millipore #MABN92, Burlington, MA, USA), and Ly-6G (Abcam #ab25377). Nuclei were visualized using DAPI staining. Imaging cytometric analysis of mouse aortas was performed using ArrayScan XTI (Thermo Fisher Scientific, Waltham, MA, USA) and FlowJo 10 software (Becton Dickinson). Two aortic tissue sections were obtained from each mouse in four experimental groups, namely DMSO, BA + DMSO, rapamycin, and BA+rapamycin, where each experimental group included three mice.

### 4.3. Expression Analysis

To analyze protein expression, frozen aortic samples were pulverized using an SK mill (Tokken, Kashiwa, Japan) and proteins extracted with RIPA buffer (Nacalai Tesque #08714-04, Kyoto, Japan). After resolving the proteins using NuPAGE gels (Invitrogen, Carlsbad, CA, USA), immunoblotting was performed with antibodies to cyclin D3 (Cell Signaling Technology #2936), p70 S6 kinase (Cell Signaling Technology #2708), phospho-p70 S6 kinase (Cell Signaling Technology #9234), phospho-Stat3 (P-Tyr705, Cell Signaling Technology #9145), Stat3 (Cell Signaling Technology #12640), Smad2 (Cell Signaling Technology #5339), phospho-Smad2 (Ser465/467, Cell Signaling Technology #3108), Jnk (Abcam #ab112501), phospho-Jnk (Thr183/Tyr185, Cell Signaling Technology #4668), tenascin-C (Immuno-Biological Laboratories #10337, Fujioka, Japan), Akt1 (Cell Signaling Technology #2938), phospho-Akt1 (Ser473, Cell Signaling Technology #4060), Akt2 (Cell Signaling Technology #3063), phospho-Akt2 (Ser473, Abcam #ab38513), SM2 (Yamasa #7601, Choshi, Japan), non-muscle myosin IIB (SMemb, Abcam #ab684), GAPDH (Millipore #MAB374), and α-tubulin (Abcam #ab7291). We performed transcriptome analyses using the SurePrint G3 Mouse Gene Expression v2 8x60K Microarray Kit (Agilent, Santa Clara, CA, USA). The dataset has been deposited in the Gene Expression Omnibus of the National Center for Biotechnology Information (accession #GSE138558). Gene enrichment analysis was performed using the Database for Annotation, Visualization, and Integrated Discovery (DAVID, https://david.ncifcrf.gov/) [42]. 

### 4.4. Gene Expression Network Analysis

For the gene expression network analysis, we used the Bayesian network algorithm and the resultant network was expressed in a two-dimensional organic layout of nodes that represent individual genes, in which the proximity of the nodes indicates the strength of the correlations [11]. The nodes are connected by edges that represent the regulatory relationships that have the directionalities of up- or down-regulation as well as which gene regulates another. This analysis is based on the assumptions that (1) gene expression in the aorta is regulated by fixed relationships among the genes, making a regulatory network structure, and (2) a gene-expression pattern in a given condition is the result of a specific pattern of regulatory network activation without a change in the network structure. To construct the structure of the gene expression network in aortic tissue, we identified a set of genes of which expressions were significantly altered by various conditions. We calculated the relationships between a pair of genes with all combinations in the gene set. If the expressions of a pair of genes are regulated always in the same, or always in the opposite direction, regardless of the condition, those genes can be regarded as tightly coupled and the correlation is high. In order to calculate the relationships, we combined the transcriptome dataset of the aortic tissue in our AD model in various conditions (Figure S5), namely *Il17a* deletion and high salt challenge (GSE116434) [21], smooth muscle-specific deletion of *Socs3* (GSE147078) [8], and rapamycin treatment in the current study (GSE138558). The gene expression changes in a given comparison of two experimental conditions were considered significant when *p* < 0.01 and the fold changes were either more than 4 or less than 0.25 (Figure S6), which identified 1,221 non-redundant genes (Figure S7, Table S2). These genes were used to construct a single gene expression network in the aortic tissue.

### 4.5. Cell Culture Experiments

Mouse aortic SMCs (#JCRB0150) were obtained from the National Institutes of Biomedical Innovation, Health and Nutrition (Tokyo, Japan) and maintained in Dulbecco’s modified Eagle’s medium (Invitrogen) with high glucose and fetal bovine serum. Confluent SMCs were serum-starved for 24 h before starting the experiments. Serum-starved SMCs were stimulated with 10 nmol/L rapamycin or DMSO as vehicle for 1 h, followed by stimulation with or without 200 nmol/mL angiotensin II and 0.2 mM BAPN for 1 h. Cellular proteins were solubilized in RIPA buffer and subjected to immunoblotting.

### 4.6. Data Acquisition and Statistical Analysis

Animals were randomly assigned to the experimental groups. The data were acquired by researchers or technicians who were blinded to the genetic modification and experimental intervention of the mice. All data are expressed as means ± standard errors. Statistical analyses were performed with GraphPad PRISM 5 (GraphPad Software, San Diego, CA, USA). When the data passed the D’Agostino and Pearson normality test and Bartlett’s test for equal variances, we performed one-way analysis of variance to compare three or more groups, followed by Bonferroni’s multiple comparison correction. For non-normal data distributions, we performed the Kruskal–Wallis test followed by Dunn’s multiple-comparison test. For discontinuous data, we performed Fisher’s exact test to evaluate the statistical significance of the observations. Significance was accepted with a two-sided *p* < 0.05.

## Figures and Tables

**Table 1:** Effect of cell cycle inhibitors on AD incidence.

AD Phenotype	BA + DMSO	BA + Gef	BA + Rapa
AD +	11	9	2
AD –	3	0	6
Total	14	9	8
Incidence (%)	78.6	100.0	25.0

*p* < 0.01 by Fisher’s exact test.

**Table 2:** Gene annotation analysis for Subnetworks #1–#3 in Figure 3.

**Subnetwork #1**			
**Annotation cluster 1 (Enrichment score: 11.77)**			
Category	Term	Gene count	*p* value
GOTERM_BP	GO:0007049, cell cycle	49	<0.001
GOTERM_BP	GO:0022402, cell cycle process	42	<0.001
GOTERM_BP	GO:0000278, mitotic cell cycle	36	<0.001
**Annotation cluster 2 (Enrichment score: 10.65)**			
Category	Term	Gene count	*p* value
GOTERM_CC	GO:0044427, chromosomal part	37	<0.001
GOTERM_CC	GO:0005694, chromosome	38	<0.001
GOTERM_CC	GO:0000228, nuclear chromosome	24	<0.001
**Annotation cluster 3 (Enrichment score: 5.91)**			
Category	Term	Gene count	*p* value
GOTERM_BP	GO:0007067, mitotic nuclear division	26	<0.001
GOTERM_BP	GO:0051301, cell division	28	<0.001
GOTERM_BP	GO:0000226, microtubule cytoskeleton organization	17	<0.001
**Subnetwork #2**			
**Annotation cluster 1 (Enrichment score: 17.04)**			
Category	Term	Gene count	*p* value
GOTERM_BP	GO:0006955, immune response	66	<0.001
GOTERM_BP	GO:0006952, defense response	66	<0.001
GOTERM_BP	GO:0002684, positive regulation of immune system process	41	<0.001
**Annotation cluster 2 (Enrichment score: 17.03)**			
Category	Term	Gene count	*p* value
GOTERM_BP	GO:0006952, defense response	66	<0.001
GOTERM_BP	GO:0009605, response to external stimulus	81	<0.001
GOTERM_BP	GO:0051707, response to other organism	43	<0.001
**Annotation cluster 3 (Enrichment score: 9.43)**			
Category	Term	Gene count	*p* value
GOTERM_BP	GO:0050729, positive regulation of inflammatory response	18	<0.001
GOTERM_BP	GO:0050727, regulation of inflammatory response	21	<0.001
GOTERM_BP	GO:0002675, positive regulation of acute inflammatory response	8	<0.001
**Subnetwork #3**			
**Annotation cluster 1 (Enrichment Score: 2.40)**			
Category	Term	Gene count	*p* value
GOTERM_BP	GO:0090066, regulation of anatomical structure size	23	<0.001
GOTERM_BP	GO:0032535, regulation of cellular component size	16	<0.005
GOTERM_BP	GO:0008361, regulation of cell size	9	<0.05
**Annotation cluster 2 (Enrichment Score: 2.06)**			
Category	Term	Gene count	*p* value
GOTERM_BP	GO:0055001, muscle cell development	11	<0.005
GOTERM_CC	GO:0044449, contractile fiber part	12	<0.005
GOTERM_BP	GO:0030239, myofibril assembly	6	<0.005
**Annotation cluster 3 (Enrichment Score: 2.06)**			
Category	Term	Gene count	*p* value
GOTERM_BP	GO:0007584, response to nutrient	11	<0.005
GOTERM_BP	GO:0031667, response to nutrient levels	18	<0.005
GOTERM_BP	GO:0009991, response to extracellular stimulus	18	<0.01

**Table 3:** Effect of the therapeutic intervention by rapamycin on the incidence of AD.

AD Phenotype	BA+DMSO Day 7	BA+DMSO Day 14	BA+Rapa Day 14
AD +	13	10	9
AD –	14	0	4
Total	27	10	13
Incidence (%)	48.1	100.0	69.2

*p* = 0.32 by Fisher’s exact test.

## Supplementary Materials

Supplementary materials can be found at https://www.mdpi.com/1422-0067/21/9/3341/s1. Figure S1: Systolic blood pressure, pulse rate, and body weight of mice, Figure S2: Rapamycin administration protocols, Figure S3: Representative immunofluorescence staining images corresponding to Figure 2, Figure S4: Representative immunofluorescence staining images for Ki67 and cell type markers, Figure S5: Pair-wise comparisons in the transcriptome data sets, Figure S6: Volcano plots for the pair-wise comparisons in the transcriptome data sets, Figure S7: The heat map representation of hierarchical clustering of AD-related genes, Figure S8: Gene expression network in AD, Figure S9: Representative immunofluorescence staining images corresponding to pStat3 staining in Figure 5A, Figure S10: Representative immunofluorescence staining images corresponding to pAkt1 and pAkt2 staining in Figure 5A, Table S1: Effect of rapamycin on AD incidence, Table S2: Genes in Subnetworks #1 – #3.

## Abbreviations

AD	aortic dissection
AngII	angiotensin II
BAPN	β-aminopropionitrile
DAPI	4’,6-diamidino-2-phenylindole
DMSO	dimethylsulfoxide
EGFR	epidermal growth factor receptor
GAPDH	glyceraldehyde-3-phosphate dehydrogenase
IGF-1	insulin-like growth factor-1
mTOR	mechanistic target of rapamycin
PI3-K	phosphatidylinositol 3-kinase
S6K	p70 S6 kinase
Smad	Sma- and Mad-related protein
SMC	smooth muscle cell
Stat	signal transducer and activator of transcription
Tgfbr	transforming growth factor β receptor
Tnc	tenascin C
