# Supplementary material for: Therapeutic Effect of Rapamycin on Aortic Dissection in Mice
Source: Int J Mol Sci. 2020 May 8;21(9):3341. doi: 10.3390/ijms21093341 (PMC7246910; doi:10.3390/ijms21093341)
Supplement: Supplementary file 1 [file ijms-21-03341-s001.pdf]

**Supplementary Materials:**

**Supplemental Table S1.** Effect of rapamycin on AD incidence.

| | **BA+DMSO** | **BA+Rapa** |
|---|---|---|
| AD + | 25 | 3 |
| AD – | 11 | 12 |
| Total | 36 | 15 |
| Incidence (%) | 69.4 | 20.0 |

*p < 0.01* by Fisher's exact test

**Supplemental Table S2.** Genes in Subnetworks #1 – #3.

| | | | | | | | | | |
|---|---|---|---|---|---|---|---|---|---|
| **Subnetwork #1** | | | | | | | | | |
| Adam12 | Adgre1 | Adm | Aldh1b1 | Aldh2 | Aqp5 | Asf1b | Asns | Atp6v1g2 | Aurka |
| Batf | Birc2 | Birc5 | Ccl2 | Ccna2 | Ccnb1 | Ccnb2 | Ccne1 | Ccnf | Cd68 |
| Cdc20 | Cdca3 | Cdca5 | Cdca8 | Cdkn2a | Cdt1 | Cenpa | Cenpe | Cenpi | Cenpk |
| Cgref1 | Chaf1b | Cidea | Creb3l3 | Cxxc4 | Diras2 | E2f1 | E2f2 | Ebi3 | Eme1 |
| Epha1 | Faim2 | Fam111a | | Fanca | Fen1 | Fes | Fn3k | Foxm1 | Fscn1 |
| Gale | Garem | Gdf6 | Gp49a | Gpr39 | Gstm1 | Has1 | Hist1h1b | Hist1h2ab | Hist1h2ag |
| Hist1h2ah | Hist1h2ak | Hist1h2an | | Hist1h2ao | | Hmga1 | Hyal1 | Ifi205 | Igf1 |
| Kif20a | Kif22 | Klhl38 | Lrr1 | Lsmem2 | Mapk11 | Mcm10 | Mcm5 | Mki67 | Mlkl |
| Murc | Mybl2 | Ncaph | Ngf | Nr4a1 | Nsl1 | Nusap1 | Optc | Osr2 | Pdzrn4 |
| Pidd1 | Plk1 | Pole | Prc1 | Prkar1a | Psat1 | Psrc1 | Ptger1 | Ptgs2 | Racgap1 |
| Rad51ap1 | Rfx2 | Rrm2 | Sapcd2 | Sgol1 | Sh3rf2 | Slc14a2 | Slc17a9 | Slc25a22 | Slc25a34 |
| Sost | Spc25 | Stmn1 | Tacc3 | Thbs1 | Tk1 | Tmtc2 | Tnfaip8l1 | Tnfrsf23 | Tpx2 |
| Traip | Trip13 | Ube2c | Uhrf1 | Vstm4 | Wisp1 | | | | |
| **Subnetwork #2** | | | | | | | | | |
| Adam11 | Adam8 | Adamts4 | | Adgre1 | Adora2b | Aif1 | Alas2 | Alox5ap | Alpl |
| Ambp | Anxa8 | Apoc2 | B4galnt1 | Batf | Bcl2a1d | Bcl3 | Begain | Bhlhe40 | Blnk |
| Bmper | Btk | Bves | C1qa | C1qb | C1qc | C5ar1 | Ccdc88b | Ccl12 | Ccl2 |
| Ccl6 | Ccl7 | Ccl8 | Ccl9 | Ccr2 | Ccr5 | Cd209a | Cd5 | Cd52 | Cd72 |
| Cfp | Ch25h | Chil3 | Clec4a1 | Clec4d | Clec4n | Cmpk2 | Col8a1 | Coro1a | Cotl |
| Csf2rb | Csf2rb2 | Cthrc1 | Ctss | Cx3cr1 | Cxcl14 | Cysltr1 | Cyth4 | Dbp | Dclk1 |
| Ddah1 | Dok2 | Dpep2 | Dusp8 | Ear10 | Ear2 | Fam180a | Fcer1g | Fcgr1 | Fcgr2b |
| Fcgr4 | Fcrls | Fes | Ffar2 | Fgd3 | Fst | Gda | Gng2 | Gp49a | Gpr132 |
| Gpr35 | Gpr65 | Grem2 | H19 | Hck | Hdac11 | Hk3 | Hmga1 | Hp | Ifi27l2a |
| Ifit3 | Ifitm6 | Ihh | Il11 | Il1b | Il33 | Il4ra | Itga6 | Itgb2 | Itgb7 |
| Jun | Kcnc4 | Kctd16 | Kng1 | Krtap11-1 | | Laptm5 | Lat2 | Lcp1 | Lgals3bp |
| Lgi3 | Lilra6 | Lilrb4 | Lpxn | Lrg1 | Lrmp | Lrrc25 | Ltb4r1 | Lyz1 | Meox1 |
| Mmp19 | Ms4a6c | Ms4a6d | Ms4a7 | Msr1 | Myo1f | Myo1g | Ncam1 | Ncf1 | Ncf4 |
| Nfam1 | Nrros | Oas1a | Oas1f | Oasl1 | Pabpc1l | Panx1 | Pappa2 | Pde10a | Pdpn |
| Pgc | Piezo2 | Pik3r5 | Pilra | Pira11 | Pira6 | Pira7 | Pirb | Pitpnm3 | Plac8 |
| Plbd1 | Pld4 | Prg4 | Ptpn6 | Ptpn7 | Ptx3 | Rab26 | Rac2 | Rbm24 | Retnla |
| Rnase2a | Rps6ka1 | S1pr3 | Saa3 | Satb1 | Selplg | Serinc2 | Serpina3g | Serpina3n | |
| Serpinb1a | | Sfmbt2 | Sh2b2 | Siglec1 | Sirpb1a | Slc13a3 | Slc15a3 | Slc16a6 | Slc2a6 |
| Slc38a11 | Slc40a1 | Slfn2 | Snx20 | Soat2 | Tcap | Timp1 | Tlr1 | Tmem173 | Tmem37 |
| Tnfaip2 | Tnfaip8l2 | Tnfrsf11b | | Tnfsf15 | Tnni2 | Tpbg | Trem2 | Treml1 | Ttc9 |
| Tubb3 | Tyrobp | Ubash3b | | Ucp2 | Ucp3 | Upp1 | Vav1 | Vsig4 | Wfdc17 |
| Wfdc21 | Wnk2 | Zbp1 | | | | | | | |
| **Subnetwork #3** | | | | | | | | | |
| Aass | Ackr4 | Acyp1 | Adcyap1 | Adra1b | Aff3 | Ak4 | Akap7 | Aldh1a2 | Aldh6a1 |
| Amd-ps5 | Anapc15 | Arid3a | Atg9b | Bloc1s3 | Cabp5 | Calcoco2 | Camsap2 | Card6 | Cat |
| Catsper4 | Ccdc172 | Cd209f | Cd209g | Cd22 | Cd300ld2 | Cd300lf | Cd300lh | Cdc27 | Cdnf |
| Cep295 | Cftr | Chat | Chst4 | Chsy3 | Clstn3 | Cmah | Cmya5 | Cnr1 | Col15a1 |
| Coro6 | Cped1 | Crispld1 | | Crnde | Csrnp3 | Cuedc1 | Cwc22 | Cyp17a1 | Cystm1 |
| Dcaf12l2 | Dcbld2 | Ddx25 | Defb28 | Dlg2 | Dmkn | Duoxa1 | Dusp7 | Dync1i1 | E4f1 |
| Egln3 | Elf4 | Fam107a | | Fam135a | Fam13c | Fam19a3 | Fam210a | Fbxo40 | Fgfr4 |
| Fmn2 | Fmr1nb | Folr1 | Fyttd1 | Gabra2 | Gata4 | Gbp6 | Gbx1 | Gclm | Git2 |
| Gmpr | Gnal | Gpr153 | Grb10 | Grifin | H1foo | H1fx | Hap1 | Hdc | Hebp2 |
| Herc2 | Ifna11 | Il18r1 | Iqcf4 | Iqcg | Iqsec3 | Klk12 | Klra1 | Klra22 | Klra7 |
| Kmt2d | Knstrn | Krt19 | Krt8 | Krtap19-4 | | Kxd1 | Lgals2 | Lhx1os | Limk1 |
| Lrrc2 | Lrrc8b | Lrrn4 | Lyplal1 | Lzts3 | Map7 | Mapt | Mast3 | Maz | Mbtd1 |
| Med1 | Micall2 | Mme | Mrgprg | Ms4a5 | Msln | Mt3 | Mthfs | Mtpn | Mybphl |

| | | | | | | | | | |
|---|---|---|---|---|---|---|---|---|---|
| Myo18b | N6amt1 | Nanog | Nav2 | Neb | Nkx2-3 | Nog | Notch1 | Nr1i3 | Oacyl |
| Olfr1500 | P2ry14 | Pabpc1l2a-ps | | Pabpc1l2b-ps | | Pabpc4 | Pabpc6 | Pak6 | Pdik1l |
| Pdzrn3 | Pif1 | Pign | Pik3c2b | Pinx1 | Pitpnc1 | Plekhh2 | Plet1 | Plp1 | Pnma2 |
| Podn | Ppapdc2 | Prex2 | Prr3 | Ptk7 | Rab26os | Rabgap1 | Rabif | Ranbp17 | Rasal3 |
| Rbm15b | Rergl | Rhox3a | Ripk2 | Rnf141 | Rnf165 | Rnf219 | Rnu2-10 | Rnu3b1 | Rpp40 |
| Rprl2 | Rs1 | Rsph4a | Rspo3 | Scn3b | Serpini1 | Sgcd | Shank2 | Shc4 | Slc25a16 |
| Slc35d1 | Slc35e2 | Slc35g1 | Slpi | Snora74a | Sowahb | Sox1 | Spata24 | Srbd1 | Src |
| Srgap3 | Srp54b | Srsf11 | Ssfa2 | St6galnac1 | | Suv39h1 | Syn3 | Tbx22 | Tcf3 |
| Tcof1 | Tdgf1 | Tgfbrap1 | | Tle4 | Tlr12 | Tmed7 | Tmem126b | Tmem170b | |
| Tmem55a | Tmem56 | Tmem59l | | Tmprss11b | | Tnc | Tnfaip8 | Triobp | Trmt6 |
| Trp63 | Trpm1 | Tulp1 | Txlnb | Ucp1 | Upk1b | Usp22 | Usp36 | Usp6nl | Usp8 |
| Utp15 | Vav2 | Vmn1r87 | | Vpreb2 | Wdr38 | Wdr54 | Wee1 | Wfdc13 | Wnt6 |
| Xk | Xpo4 | Yy2 | Zdhhc2 | Zfhx2 | Zfp691 | Zfp740 | Zfp790 | Zswim4 | |

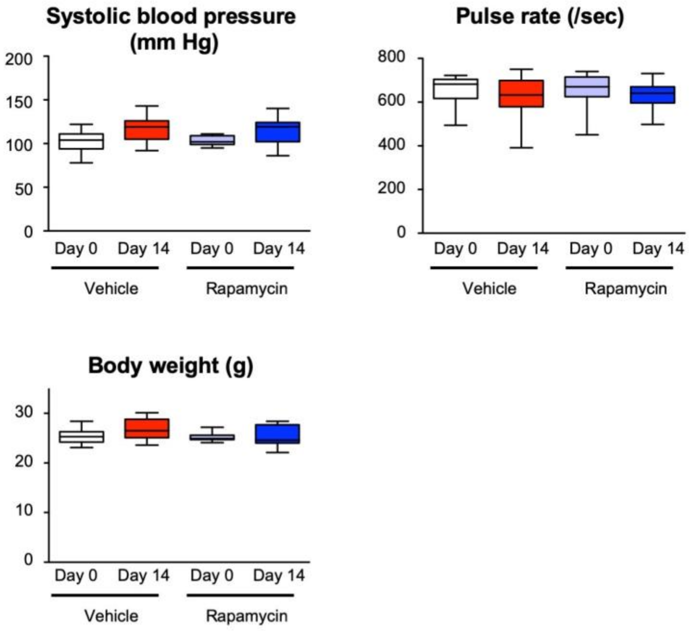


**Supplemental Figure S1.** Systolic blood pressure, pulse rate, and body weight before and after 14 days of BAPN+AngII challenge, with and without rapamycin treatment.

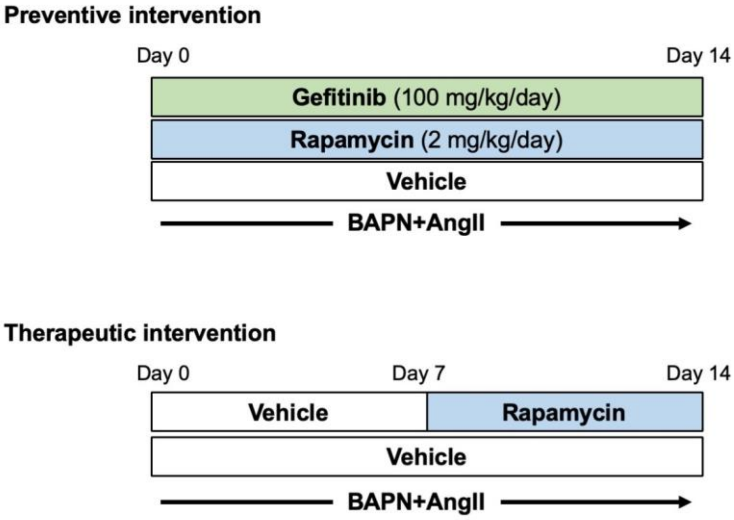


**Supplemental Figure S2.** Rapamycin treatment was performed either in the preventive intervention or in the therapeutic intervention. In the preventive intervention, intraperitoneal administration of rapamycin or vehicle (DMSO) was started on the day of the implantation of BAPN and AngII pumps, and continued throughout the 14-day observational period. In the therapeutic intervention, both the vehicle group and the rapamycin group received vehicle on the day of BAPN pump and AngII pump

implantation and continued to day 7, when rapamycin administration was started, and continued for the rest of the observational period.

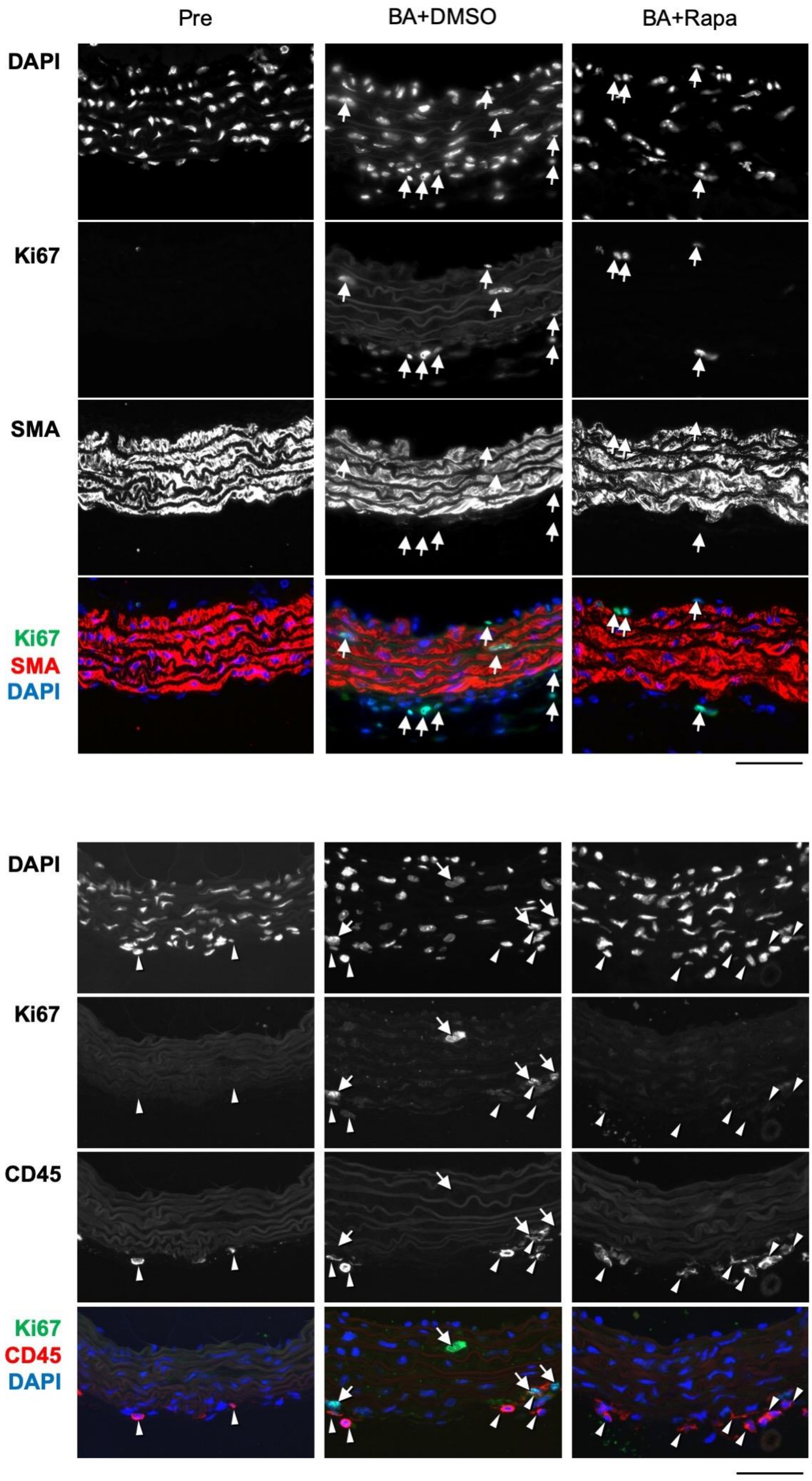


**Supplemental Figure S3.** Representative immunofluorescence staining images corresponding to Figure 2. Separate images for DAPI, Ki67, and SMA or CD45 stainings are shown in gray scale. Merged images are also shown in color. Arrows indicate Ki67-positive nuclei. Arrowheads indicate CD45-positive cells. Scale bars: 50 μm.

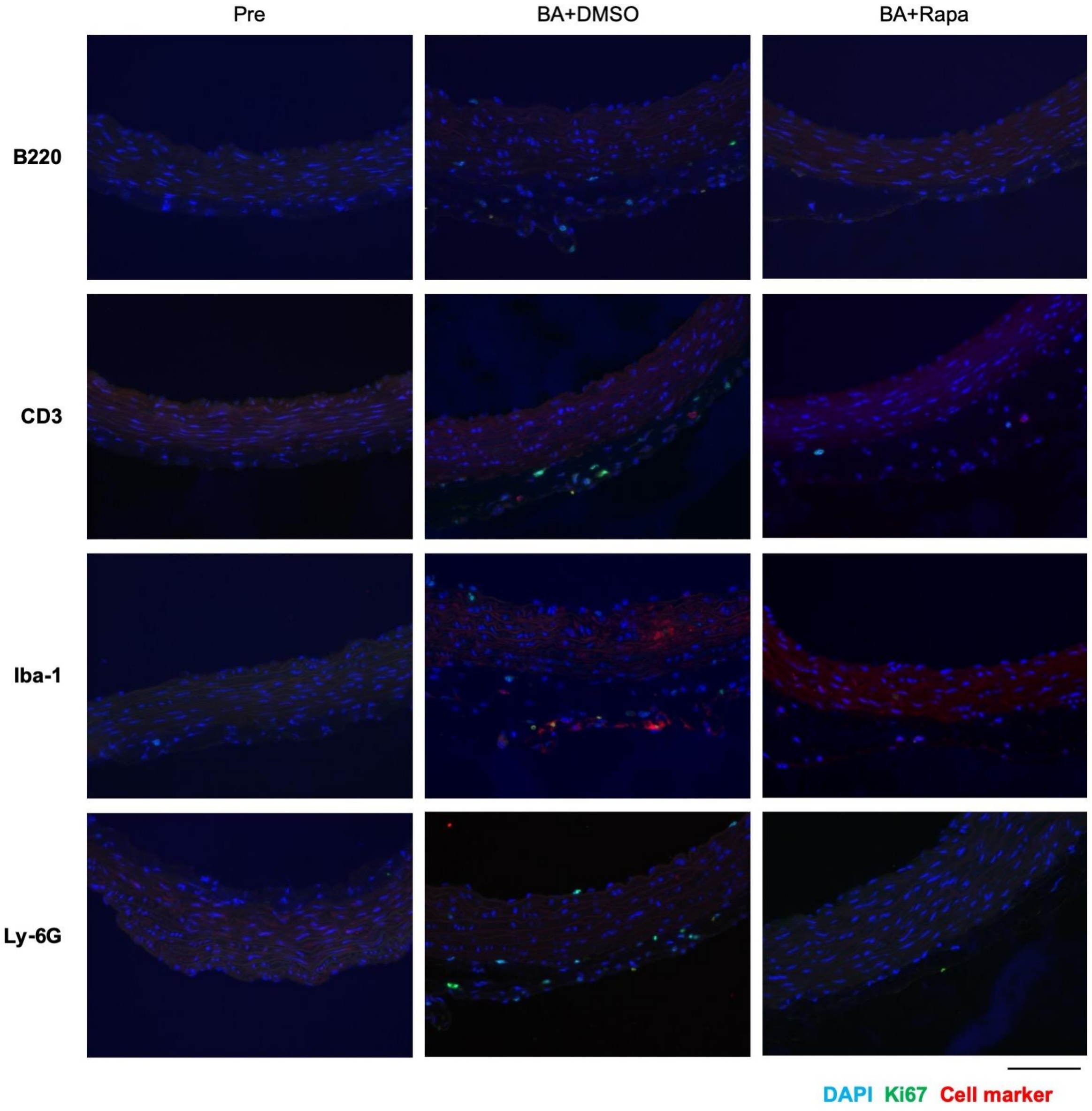


**Supplemental Figure S4.** Representative immunofluorescence staining images for Ki67 and cell type markers for B cells (B220), T cells (CD3), monocyte/macrophages (Iba-1), and neutrophils (Ly-6G). BAPN+AngII challenge was applied for 3 days with or without rapamycin treatment. Scale bar: 50 µm.

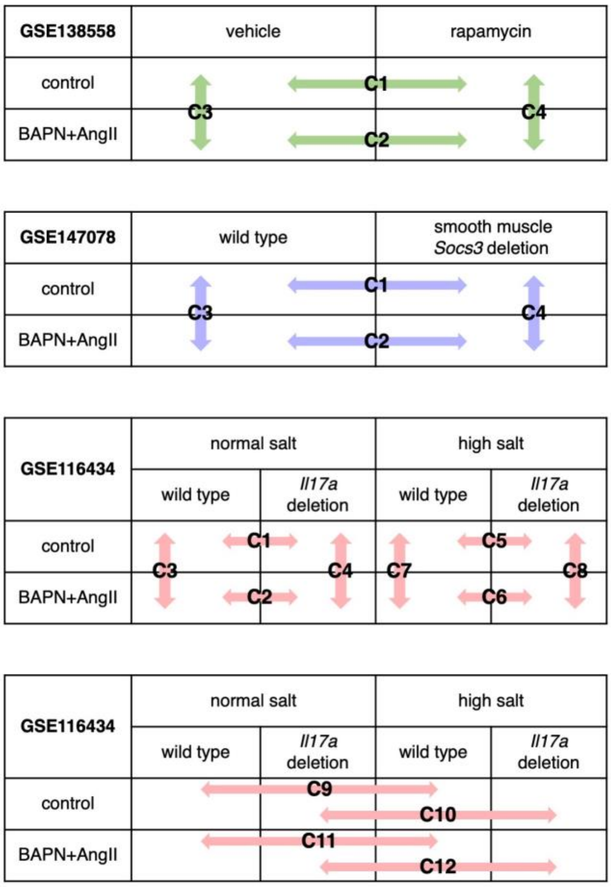


**Supplemental Figure S5.** The diagrams depict, by double arrows, the pair-wise comparisons in three transcriptome data sets; GSE116434 [21], GSE147078 [8], and GSE138558. All of the samples were thoracic aortas of the same AD model as the current study, with different experimental interventions. GSE116434: normal vs. high salt challenge and wild type vs. *Il17a* deletion. GSE147078: wild type vs. smooth muscle-specific *Socs3* deletion. GSE138484: vehicle vs. rapamycin. Each experimental group contained three biological replicates (mice), and in total 48 aortic samples were used to construct the gene expression network in the AD model. Twelve comparisons (C1–C12) for GSE116434, and 4 comparisons (C1–C4) each for GSE147078 and GSE138558 were made to extract the genes with significant changes in expression.

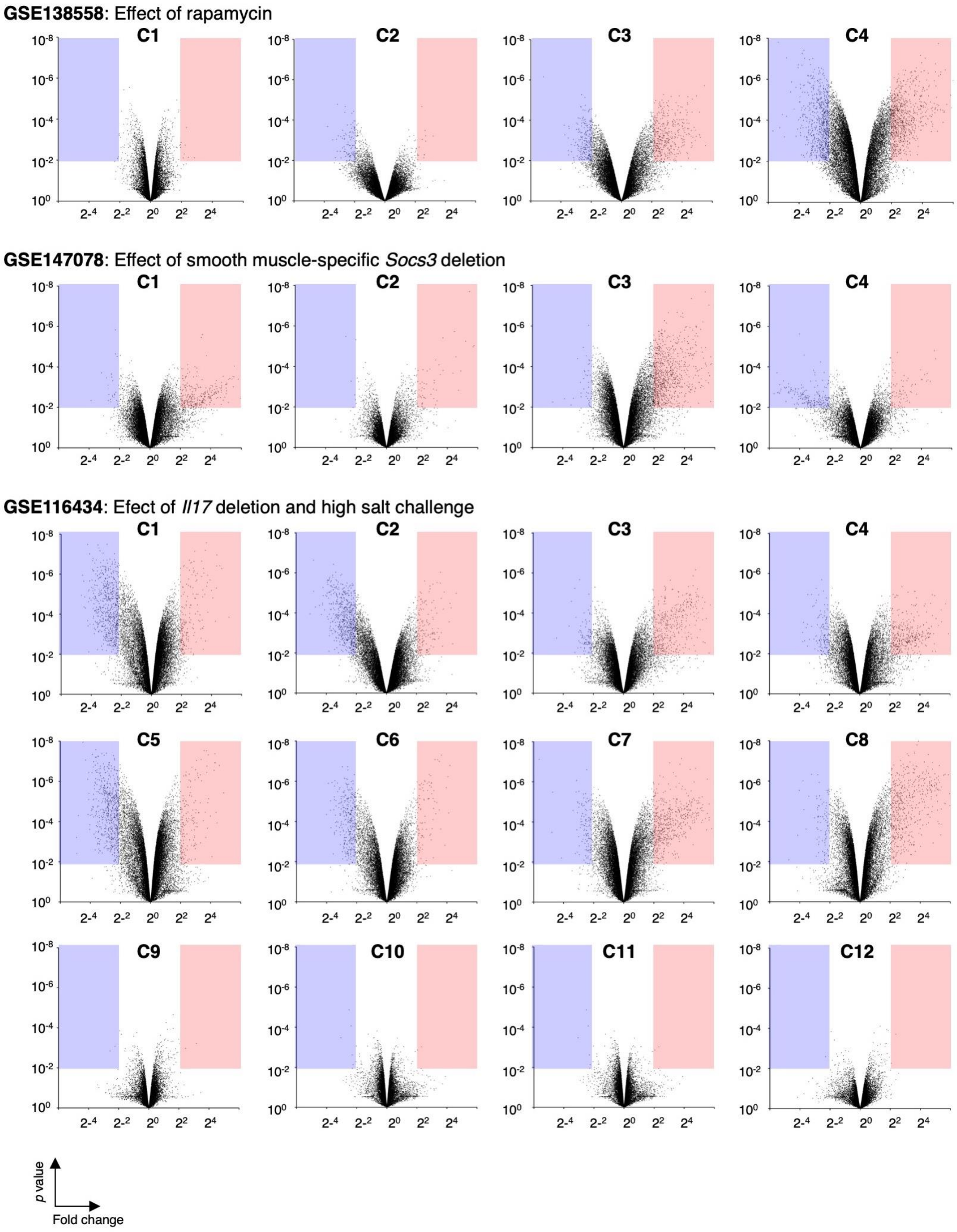


**Supplemental Figure S6.** Volcano plots for the comparisons in GSE116434 [21], GSE147078 [8], and GSE138558, as depicted in Supplemental Figure S3. The changes in gene expressions were considered significant when the fold changes were more than 4 (induced) or less than 0.25 (suppressed), and $p$ values were less than 0.01. Induced genes and suppressed genes are indicated by red and blue shading, respectively.

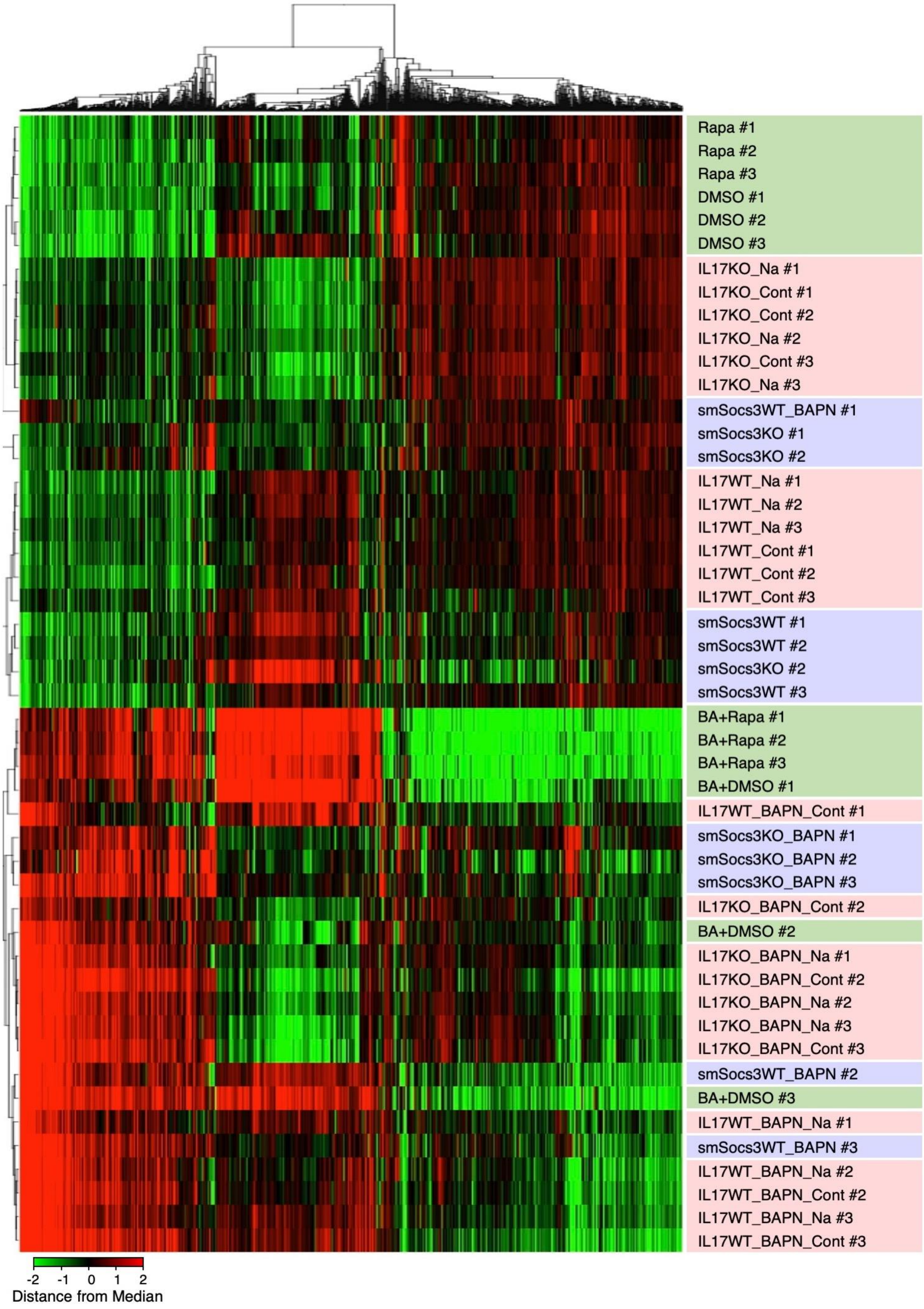


**Supplemental Figure S7.** The heat map representation of hierarchical clustering of the 1221 AD-related genes extracted from datasets GSE116434 [21], GSE147078 [8], and GSE138558. The sample names are color coded in red for GSE116434, blue for GSE147078, and green for GSE138558. These data were used for the gene network analysis in Figure 3.

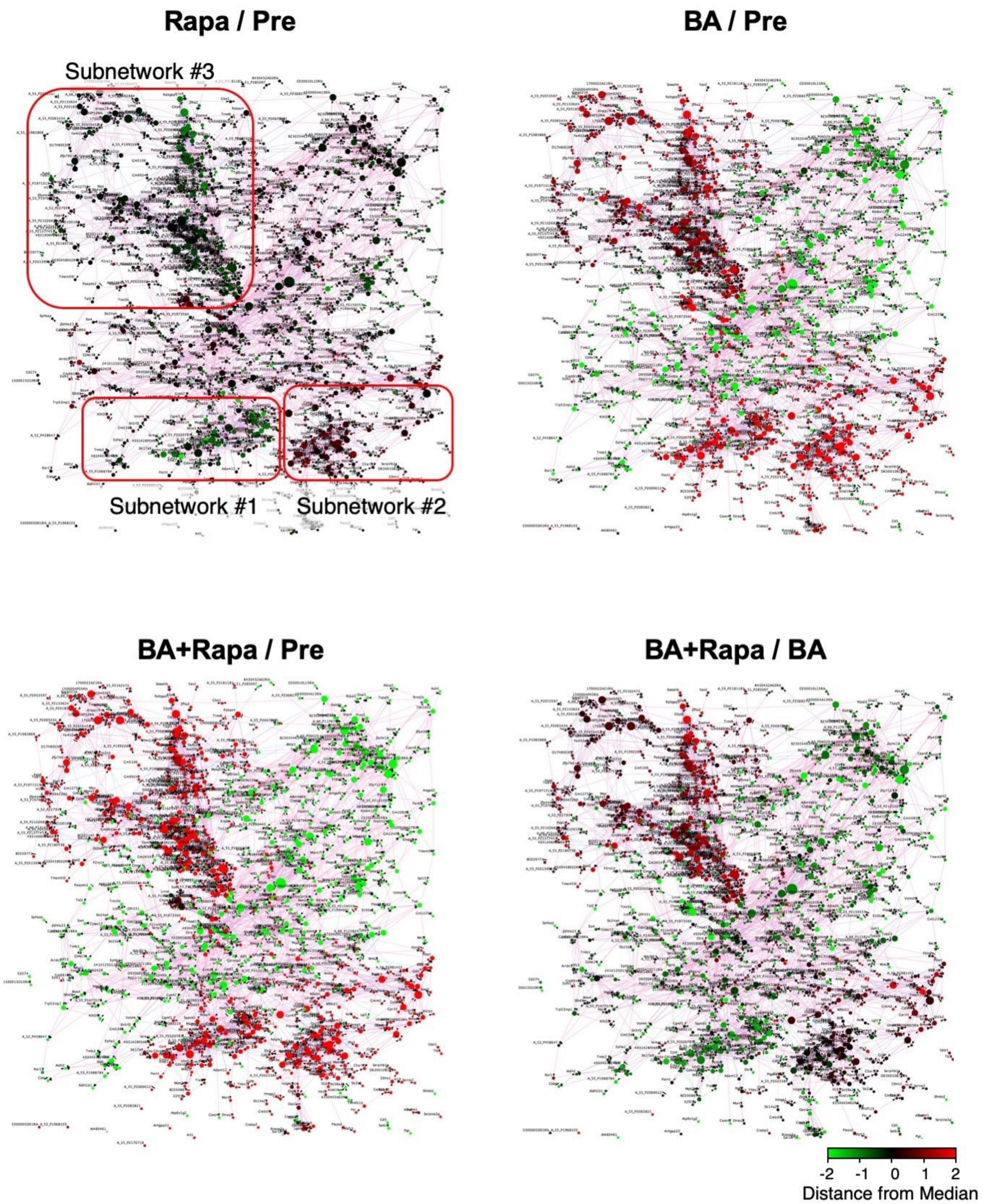


**Supplemental Figure S8.** Gene expression network in AD. Organic plots of the gene expression according to Bayesian network analysis. Thoracic aortas were obtained with or without 3 days of BAPN+AngII challenge. The comparisons shown are between no intervention and rapamycin alone (Pre vs. Rapa), Pre and BAPN+AngII challenge (Pre vs. BA), Pre and BAPN+AngII challenge with rapamycin treatment (Pre vs. BA+Rapa), and BAPN+AngII challenge without and with rapamycin treatment (BA vs. BA+Rapa). Each node in the organic plots represents a single gene that is color coded for induction (red) or suppression (green) in a given comparison.

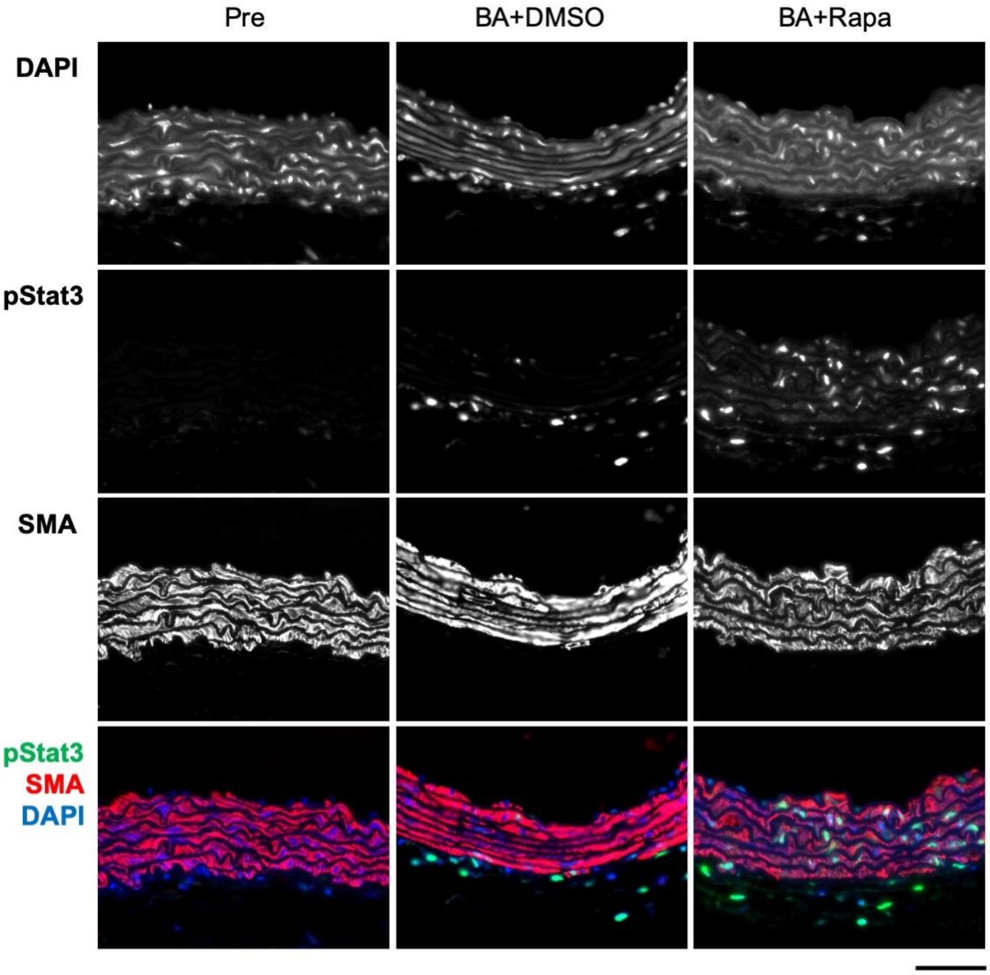


**Supplemental Figure S9.** Representative immunofluorescence staining images corresponding to pStat3 staining in Figure 5A. Separate images for DAPI, pStat3, and SMA staining are shown in gray scale. Merged images are also shown in pseudo color: blue, green, and red for DAPI, pStat3, and SMA, respectively. Scale bar: 50 µm.

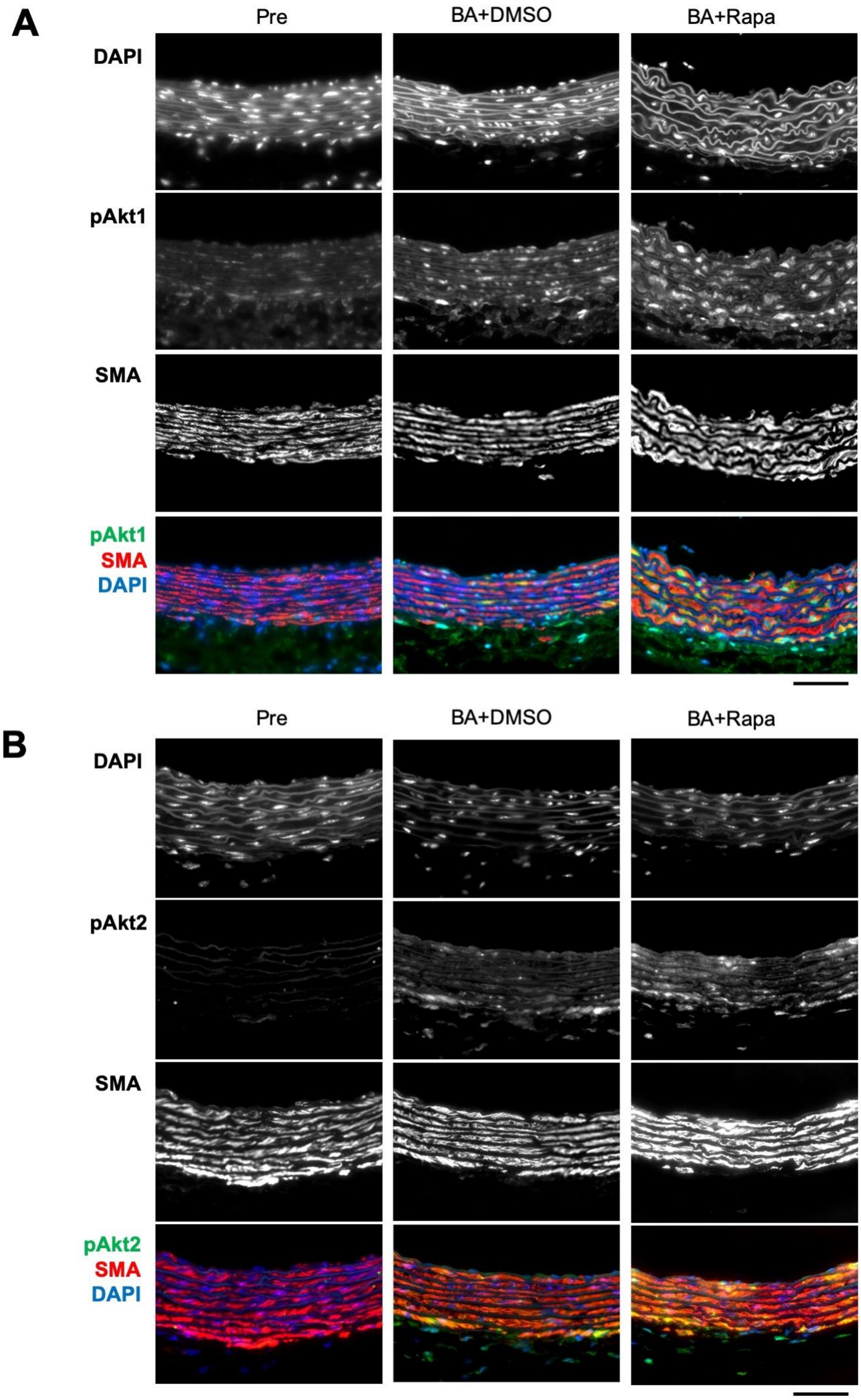


**Supplemental Figure S10.** Representative immunofluorescence staining images corresponding to pAkt1 and pAkt2 staining in Figure 5A. Separate images for DAPI, pAkt1 or pAkt2, and SMA staining are shown in gray scale. Merged images are also shown in pseudo color: blue for DAPI, green for pAkt1 or pAkt2, and red for SMA. Scale bar: 50 µm.
